# An interdisciplinary multimodal integrative healthcare program for depressive and anxiety disorders

**DOI:** 10.3389/fpsyt.2023.1113356

**Published:** 2023-06-23

**Authors:** Jaap Wijnen, Nicole Louise Gordon, Geert van 't Hullenaar, Marc Lucas Pont, Marciano Wilhelmina Henricus Geijselaers, Jessica Van Oosterwijck, Jeroen de Jong

**Affiliations:** ^1^Intergrin Academy, Geleen, Netherlands; ^2^Spine, Head and Pain Research Unit Ghent, Department of Rehabilitation Sciences, Faculty of Medicine and Health Sciences, Ghent University, Ghent, Belgium; ^3^Pain in Motion International Research Group, Brussels, Belgium; ^4^Center for InterProfessional Collaboration in Education Research and Practice (IPC-ERP UGent), Faculty of Medicine and Health Sciences, Ghent University, Ghent, Belgium

**Keywords:** depression, anxiety, multimodal intervention, behavioral therapy, interdisciplinary, transdiagnostic, psychosomatic

## Abstract

**Objective:**

Although multimodal interventions are recommended in patients with severe depressive and/or anxiety disorders, available evidence is scarce. Therefore, the current study evaluates the effectiveness of an outpatient secondary care interdisciplinary multimodal integrative healthcare program, delivered within a transdiagnostic framework, for patients with (comorbid) depressive and/or anxiety disorders.

**Methods:**

Participants were 3,900 patients diagnosed with a depressive and/or anxiety disorder. The primary outcome was Health-Related Quality of Life (HRQoL) measured with the Research and Development-36 (RAND-36). Secondary outcomes included: (1) current psychological and physical symptoms measured with the Brief Symptom Inventory (BSI) and (2) symptoms of depression, anxiety, and stress measured with the Depression Anxiety Stress Scale (DASS). The healthcare program consisted of two active treatment phases: main 20-week program and a subsequent continuation-phase intervention (i.e., 12-month relapse prevention program). Mixed linear models were used to examine the effects of the healthcare program on primary/secondary outcomes over four time points: before start 20-week program (T0), halfway 20-week program (T1), end of 20-week program (T2) and end of 12-month relapse prevention program (T3).

**Results:**

Results showed significant improvements from T0 to T2 for the primary variable (i.e., RAND-36) and secondary variables (i.e., BSI/DASS). During the 12-month relapse prevention program, further significant improvements were mainly observed for secondary variables (i.e., BSI/DASS) and to a lesser extent for the primary variable (i.e., RAND-36). At the end of the relapse prevention program (i.e., T3), 63% of patients achieved remission of depressive symptoms (i.e., DASS depression score ≤ 9) and 67% of patients achieved remission of anxiety symptoms (i.e., DASS anxiety score ≤ 7).

**Conclusion:**

An interdisciplinary multimodal integrative healthcare program, delivered within a transdiagnostic framework, seems effective for patients suffering from depressive and/or anxiety disorders with regard to HRQoL and symptoms of psychopathology. As reimbursement and funding for interdisciplinary multimodal interventions in this patient group has been under pressure in recent years, this study could add important evidence by reporting on routinely collected outcome data from a large patient group. Future studies should further investigate the long-term stability of treatment outcomes after interdisciplinary multimodal interventions for patients suffering from depressive and/or anxiety disorders.

## 1. Introduction

Depressive and anxiety disorders are the most common mental health disorders worldwide (1, 2). The Diagnostic and Statistical Manual of mental disorders, fifth edition (DSM-5) considers depressive and anxiety disorders as two separate diagnostic categories (3), and no longer includes a mixed subsyndromal anxiety-depressive diagnostic category as was the case in earlier versions of the DSM (4). Moreover, an increasing number of studies questions the independence of depression and anxiety, by providing evidence of: (1) shared genetic risk across the internalizing disorders (5–7), (2) high similarities in neurocircuit disruption across mental disorders, including depressive and anxiety disorders (8, 9), (3) a more parsimonious structure to psychopathology (i.e., overarching ‘p-factor’) as compared to current nosologies (i.e., DSM-5) (10, 11), and (4) comparable responses to the same treatment across different mental disorders (12). Furthermore, a study by Choi et al. (13) highlighted that 45–67% of patients with a major depressive disorder (MDD) meet criteria for at least one comorbid anxiety disorder and 30–63% of patients with anxiety disorders meet criteria for a comorbid MDD. Recent evidence from Shevlin et al. (14) concludes that co-occurring anxiety and depression may even be more common than ‘pure’ depression or anxiety disorders. In addition to high comorbidity, considerable heterogeneity in symptom presentation within diagnostic categories also poses a serious problem.

These findings emphasize the need to shift away from a ‘traditional diagnostic approach’, in which interventions are predominantly focused on symptom level and where the majority of interventions are tailored toward single diagnosis, generally ignoring comorbid conditions or symptoms that fall outside diagnostic classifications (15, 16). Instead, a shift is needed toward a ‘transdiagnostic approach’ (15), acknowledging that predisposing, precipitating and perpetuating factors implicated in mental health are not specific to particular diagnosis, but operate across traditional diagnostic categories (16–18). It is therefore important to focus on the underlying mechanisms responsible for the development and maintenance of mental disorders, cutting across multiple diagnostic categories (19, 20). With regard to treatment, a transdiagnostic intervention would apply the same underlying treatment principles across a wide range of diagnosed mental disorders, without adjusting treatment to specific diagnosis (18, 21). Indeed, a growing body of research is supporting the effectiveness of transdiagnostic interventions for individuals experiencing depression and anxiety symptoms (16, 22–24).

A remaining question concerns what treatment content constitutes best-evidence for depressive-and anxiety disorders, regardless whether an intervention is delivered within a transdiagnostic framework. Current best evidence for treatment in depressive-and anxiety disorders generally support the use of: cognitive behavioral therapy (CBT), third-wave CBT (e.g., acceptance and commitment therapy, mindfulness-based cognitive therapy, etc.), psychotherapy, relaxation, exposure therapy, biological interventions and physical activity/exercise therapy (25–29). Highest quality evidence is generally found for the use of CBT (25, 29, 30). Most of the aforementioned recommendations are based on studies investigating treatment effects of mono-interventions in patients with single diagnoses (31). However, especially in more severe cases of (comorbid) depressive and anxiety disorders, it is important to not only target one aspect of the mental disorder (i.e., cognitions/behavior in CBT), while ignoring other aspects (i.e., somatic aspects, targeted during for example exercise therapy). This emphasizes the need for the use of more holistic interventions, targeting multiple aspects of depressive and anxiety disorders.

Interdisciplinary multimodal interventions could be a suitable alternative and use at least two different therapeutic interventions with different mechanisms of action (i.e., multimodality) and are delivered by a team of healthcare professionals from at least two different professions, with a common treatment philosophy and shared therapeutic aims and treatment goals (i.e., interdisciplinary) (32–34). To date, few studies support the use of multimodal interventions in this patient group. Nonetheless, the guideline for depression from the National Institute for Health and Care Excellence (i.e., NICE) in the United Kingdom (UK) recommends multimodal interventions for patients with severe depression unresponsive to previous treatments (35). Also the UK guidelines for anxiety disorders (36, 37) recommends multimodal interventions only in severe cases of anxiety. Despite the fact that multimodal interventions are recommended in more severe cases of depressive and anxiety disorders, only a few studies have published results on the efficacy (38, 39) or effectiveness (31, 40–45) of these interdisciplinary multimodal interventions. Four studies focused on populations with severe depressive disorders as single diagnoses (39, 42, 44, 45). Only five studies evaluated interdisciplinary multimodal interventions in more realistic patient samples with comorbid depression and anxiety disorders (31, 38, 40, 41, 43). These studies report remission rates post-treatment for depressive symptoms ranging from 17% (38) to 50% (31). Even less is known about sustainability of treatment results after multimodal interventions in comorbid depression and anxiety, with only one study (40) reporting long-term (i.e., 6 months) stability of treatment results. In conclusion, more research is warranted to investigate the short and long-term effects of interdisciplinary multimodal interventions in populations with comorbid depressive and anxiety disorders.

The aim of this study was to evaluate the effectiveness of an outpatient secondary care interdisciplinary multimodal integrative healthcare program, delivered within a transdiagnostic framework, for patients suffering from (comorbid) depressive and/or anxiety disorders. The healthcare program consisted of two treatment phases: a main 20-week program and a 12-month relapse prevention program (RPP). The primary outcome was health-related quality of life (HRQoL) and secondary outcomes were psychological and physical symptoms. HRQoL is the main variable of interest since the evaluated interdisciplinary multimodal healthcare program has a ‘recovery-oriented’ approach, meaning that recovery of daily functioning and perceived quality of life is the central focus within the program and recovery of somatic and/or psychiatric symptoms is a secondary aim. The healthcare program is expected to increase HRQoL and to a lesser extent to reduce psychological/physical symptoms. In addition, we expect further improvements in HRQoL but not in psychological and physical symptoms, after completion of the 12-month RPP.

## 2. Materials and methods

### 2.1. Study design and setting

The current study uses a descriptive study design (i.e., case series), without comparator group, in order to evaluate the effectiveness of an interdisciplinary multimodal integrative healthcare program for patients with depressive and/or anxiety disorders provided at “Premium Healthcare Interventions” (PHI), a specialized secondary care mental healthcare center with branches in seven different cities across the Netherlands.

### 2.2. Participants

All patients referred to the healthcare program by general practitioners, occupational physicians and medical specialists between January 2017 and May 2022 were considered for study participation based on the following inclusion and exclusion criteria. The main inclusion criterion was the presence of a depressive or anxiety disorder as primary disorder according to the DSM-5 (3), diagnosed by a psychologist or psychiatrist. Exclusion criteria were: (1) an ongoing personal injury lawsuit, (2) ongoing social security issues (i.e., incapacitated, unemployment benefits, etc.), (3) other psychological or somatic condition that is predominant to depressive or anxiety disorder, (4) insufficient proficiency of Dutch/English language in order to understand therapy content, (5) insufficient intellectual abilities in order to understand therapy content, (6) under the age of 18. All eligible patients were asked to provide permission to use their anonymized data for research purposes by providing written consent. The study complies with the Declaration of Helsinki and the Medical Ethics Review Committee of Maastricht University Medical Center decided that this study does not need a full review (register number: 2022–3266) according to the Dutch Medical Research with Human Subjects Law (i.e., Wet Medisch-wetenschappelijk onderzoek met mensen, WMO), because it concern a descriptive study that used anonymized data from medical records.

### 2.3. Procedure

The healthcare program consisted of three phases: intake procedures (i.e., phase 1), the main 20-week outpatient healthcare program (i.e., phase 2) and a 12-month RPP (i.e., phase 3). Data was collected through questionnaires at the following time points: start of phase 2 (i.e., before start 20-week program: T0), halfway phase 2 (i.e., halfway 20-week program: T1), end of phase 2 (i.e., after 20-week program: T2) and at the end of phase 3 (i.e., end of 12-month RPP: T3). Eligible patients received an explanation about the healthcare program during an information session prior to the intake procedures (i.e., before start of phase 1). Subsequently, patients decided whether or not to start the intake procedures (phase 1), which included the completion of several standardized questionnaires and psychological and physical assessments, performed by a coordinating practitioner, various psychologists and a physical therapist. The assessments are focused on identifying predisposing, perpetuating and maintaining factors implicated in the mental health problem and cover the following areas: (1) Psychosomatics: emotion regulation, body awareness, etc., (2) Lifestyle: social participation, sleep, nutrition, etc., (3) Cognition and behavior: coping, maladaptive thoughts and behavior, etc., (4) Somatic: physical well-being, energy management, etc. Based on the assessments and questionnaire outcomes, a coordinating practitioner (i.e., psychiatrist, clinical/health care psychologist) decided whether individual patients were eligible for the healthcare program. After mutual agreement to the treatment plan, the main 20-week outpatient intervention (i.e., phase 2) was started. After completion of phase 2, patients were free to choose whether or not to participate in a subsequent 12-month RPP (phase 3).

### 2.4. Treatment program

The healthcare program provided at the specialized secondary mental healthcare center (i.e., PHI) is characterized by an interdisciplinary, multimodal and integrative approach for patients with depressive and/or anxiety disorders. The healthcare program is best described as a multimodal cognitive behavioral therapy and combines the use of different therapeutic methods, based on the problems, needs and unique circumstances of each individual client. The basic principles of behavioral therapy (46) are central to the healthcare program and treatment techniques that are being used can be justified in an adequate behavioral therapeutic process, starting with a functional analysis of problem behavior (46–48) and resulting in an individualized treatment plan. Other important elements within the healthcare program are: (1) a modular approach (15), where evidence-based elements can be delivered flexible and tailored toward the individual, based on specific underlying biopsychosocial risk, protective and/or maintaining factors of the mental health problem; (2) intensive, time-bound intervention program (49, 50); (3) an emphasis on the biopsychosocial model (51); (4) ‘shared decision making’ with collaborative goal-setting (52, 53); (5) a ‘recovery-oriented’ approach, in which patients are encouraged to cope autonomously with changing physical, emotional and social challenges (54–57); (6) a focus on relapse prevention, through the incorporation of a 12-month RPP as part of the intervention (58, 59) and (7) ‘blended care’ (60), characterized by a combination of online and offline therapy, with the support of an e-health environment.

The healthcare program is led by an interdisciplinary team consisting of psychologists, physical therapists and a coordinating practitioner (i.e., psychiatrist, clinical/health care psychologist) (see Supplementary Table S1 for detailed provider information). Individual patients are assigned to an interdisciplinary treatment team consisting of three psychologists and a physical therapist, supervised by a coordinating practitioner. Each of the three psychologists within the treatment team has a different focus, namely: (1) the relationship between cognitive, behavioral, emotional, somatic and environmental factors that are proposed to maintain the self-perpetuating cycle of symptoms, distress and disability, (2) a focus on psychosomatics (i.e., reciprocity of body and mind), (3) focus on associations in unhealthy lifestyle behaviors and symptoms/disability. Physical therapists mainly focus on the somatic symptoms/bodily dysfunctions associated with the diagnosed mental disorder(s). The main 20-week outpatient healthcare program (phase 2) had a direct total treatment time of approximately 75 hours, consisting of 3 to 4 sessions per week with a duration of 1 hour. Phase 2 of the healthcare program consisted of five modules of approximately 4 weeks, covering the following themes: (1) Introduction, goal formulation, case conceptualization and (psycho)-education, (2) Recognition of maladaptive behavioral patterns and thoughts, emotion regulation, body awareness, mentalization and behavioral activation, (3) Application of newly learned skills in personal context, behavioral experiments and exposure, (4) Strengthening the capacity to cope/manage with physical, emotional and social challenges, encouragement for behavioral change, (5) Future goals, sustainability of treatment results and relapse prevention (see Supplementary Table S2 for more details). Within these modules, appropriate evidence-based interventions were selected, including: (1) Second and third-wave generation CBTs, (2) Psychosomatic interventions with a main focus of reciprocity of body and mind; (3) Operant-based interventions (61); (4) Body-related mentalization interventions (62); (5) Relaxation therapies/stress management; (6) Exercise therapy, with a focus on the somatic symptoms/bodily dysfunctions associated with the diagnosed mental disorder(s), and (7) Lifestyle interventions. Phase 2 was followed by a subsequent 12-months ‘RPP’ (phase 3), with a main focus on sustainability of treatment results through the encouragement of self-management and patient autonomy. The program consisted of e-health modules (delivered through an online platform) and/or several treatment sessions with members of the interdisciplinary team, either face-to-face or through e-communication. The content of the e-health modules and treatment sessions were always based on the main themes covered during phase 2. The combined ‘direct’ (i.e., treatment sessions with healthcare professional) and ‘indirect’ (i.e., e-health modules) treatment time over phase 3 is approximately 10 hours over a period of 12 months. The entire healthcare program (i.e., T0 to T3) is a blended treatment which combines face-to-face contact with online interventions through the support of an e-health environment. Through the e-health environment patients were able to use psycho-educative programs, e-health modules, exercises and videos tailored by the treatment team for each individual patient.

### 2.5. Outcome measures

#### 2.5.1. RAND-36

General health and HRQoL was assessed by using the Dutch version of the Research and Development-36 (RAND-36) (63), which is the equivalent of the 36-Item Short Form Health Survey with the addition of one extra subscale, i.e., ‘health change’. The Dutch version of the RAND-36 consists of 36 items distributed across nine domains of which subscale scores can be calculated, i.e., physical functioning, role limitations due to physical health problems, role limitations due to emotional problems, general mental health, social functioning, bodily pain, vitality, general health perception and health change. Each domain is scored from 0 to 100, with higher scores indicating better health. In addition, the Physical Component Score (PCS) and Mental Component Score (MCS) can be calculated by using recommended scoring algorithms (64), with scores also ranging from 0 to 100. The PCS primarily reflects physical aspects of HRQoL and the following subscales contribute most to scoring of the PCS: physical functioning, bodily pain and role limitations due to physical health problems. The MCS primarily reflects mental aspects of HRQoL and the following subscales contribute most to scoring of the MCS: general mental health, role limitations due to emotional problems and social functioning (64). Good reliability has been reported with internal coefficients ranging from 0.71–0.93 (65). Several studies also support the validity of the RAND-36 by reporting adequate convergent validity and discriminant validity (65–67). The RAND-36 has been widely used to measure HRQoL in different patient groups, including in patients suffering from mental disorders (68, 69). PCS, MCS and RAND-36 subscales, were used as primary outcome measures in the current study.

#### 2.5.2. BSI

To assess current psychological and physical symptoms the current study used the Dutch version of the Brief Symptom Inventory (BSI) which is a multidimensional self-report inventory (70) and shorter version to the Symptom Checklist 90 (71, 72). The Dutch version of the BSI consists of 53 items with descriptions of symptoms on the following nine dimensions of which subscale scores can be calculated, i.e., somatization, cognitive problems, interpersonal sensitivity, depression, anxiety, hostility, phobic anxiety, paranoid ideation and psychoticism (i.e., 0–4 score range on all subscales). In addition, the BSI includes the Global Severity Index (GSI), which ranges from 0 to 4 and reflects the average intensity of all symptom subscales combined. Participants have to indicate on a five-point rating scale to what extent they have experienced symptoms over the past week. For each subscale, raw scores can be calculated by dividing the sum of all items for a dimension by the number of items within that subscale. The GSI is calculated by summing the scores on all nine dimensions (and scores of additional individual items) and dividing it by the total number of responses. Higher scores on the BSI reflect more severe psychopathology. Sufficient reliability has been reported with internal consistency coefficients ranging from 0.71–0.83 (70, 73, 74). Several studies also support the validity of the BSI by reporting adequate convergent validity and predictive validity (70, 74–79). The GSI and BSI subscales were used as secondary outcome measures in the current study.

#### 2.5.3. DASS

Symptoms of depression, anxiety and stress were measured by using the Dutch version (80) of the ‘Depression Anxiety Stress Scales’ (DASS) (81, 82). The DASS consists of 42 items divided over 3 subscales: depression, anxiety and stress (i.e., 14 items for each subscale). Each subscale is scored from 0 to 42, with higher scores indicating higher severity of depression, anxiety and/or stress. Respondents have to indicate on a 4-point Likert scale to what extent statements apply to them over the past week, (i.e., 0 = did not apply to me at all and 3 = applied to me very much, or most of the time). Sufficient reliability has been reported with internal consistency coefficients ranging from 0.84–0.98 (81, 83–86). Adequate convergent and discriminant validity is also reported in non-clinical and clinical samples (80, 84–87). DASS scores for depression, anxiety and stress were used as secondary outcomes in the current study.

### 2.6. Statistical analysis

Statistical analysis were performed with two Python software programs: Statsmodels (version 0.13.2) (88) and SciPy (version 1.8.0) (89). The significance level was set at 0.05 and normality was tested with the D’Agostino-Pearson omnibus test (90, 91) (see Supplementary materials, section 2.3 for python code of normality testing).

Because of unbalanced data (i.e., unequal numbers of observations per time point) mixed linear model (MLM) analysis for longitudinal data was performed by using Statsmodels (88). Separate MLMs were constructed for each dependent variable (i.e., RAND-36, BSI and DASS (sub)scales). The fixed effect added to the model as a first level predictor was ‘time’ (i.e., representing the four measurement phases: T0, T1, T2, and T3). No second level predictors were added as fixed effects to the model. For random effects, Statsmodels automatically selects the best fitting random effects covariance matrices for each MLM. In addition, random intercepts and random slopes were tested. The construction of MLM with Statsmodels was based on recommendations from previous research (88, 92). See Supplementary materials (Supplementary Data, section 2.1) for an archive link to the Statsmodels website for an explanation on how to perform an MLM in Statsmodels. In addition, python codes for the construction of the MLMs in the current study were also added to the Supplementary materials (Supplementary Data, section 2.2). For MLMs with a main effect of time, post-hoc pairwise comparisons of dependent variable means were carried out between subsequent time points, adjusting for multiple comparison using Bonferroni corrections (93) by using SciPy (89). In case of normally distributed data a dependent t-test for paired samples was used (89, 94), and for non-normal distributed data the Wilcoxon signed rank test was used (89, 95). The python code of the post-hoc pairwise comparisons are also added to the Supplementary materials (Supplementary Data, section 2.3). In order to visualize the change over time of BSI, RAND-36 and DASS (sub)scales, lines were connected between subscale averages at each time point (i.e., T0, T1, T2, and T3) (Supplementary Figures S1–S3).

Effect size calculation was done based on the independent sample and inequal sample size variant of Cohen’s *d* as described by (96). A ‘d’ value around 0.01 is described as very small, 0.20 as small, 0.50 as medium (moderate), 0.80 as large, 1.20 as very large and near 2 as huge (97, 98). Cohen’s *d* was calculated with the following formula: $d=\frac{{\bar{X}}_{post}-{\bar{X}}_{pre}}{s^{*}}$ with the pooled standard deviation $s^{*}$ defined as $s^{*}=\sqrt{\frac{\sum \left(X_{pre}-{\bar{X}}_{pre}\right)+\sum \left(X_{post}-{\bar{X}}_{post}\right)}{n_{pre}+n_{post}-2}}$ . In addition, python codes for effect size calculation as applied in the current study were also added in the Supplementary materials (Supplementary Data, section 2.4).

Minimal clinically important difference (MCID) was calculated for RAND-36 scores by using ‘multiples of standard deviation’ (i.e., 0.5*SD) as described by Norman et al. (99). The statistical analysis described here were also applied previously to another patient sample by the same research group [see Wijnen et al. (100)].

## 3. Results

### 3.1. Patient characteristics

**Figure 1:**
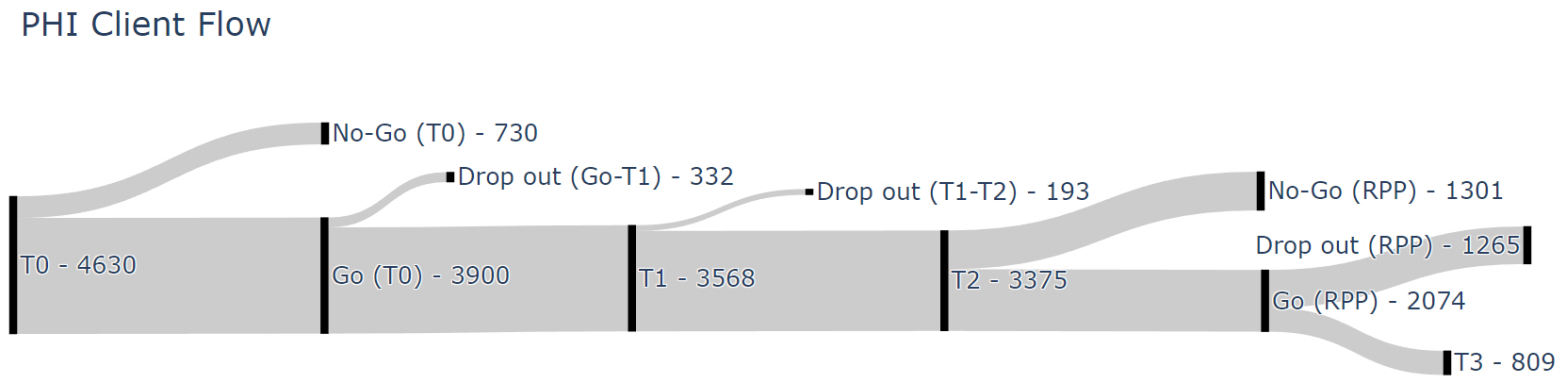
Participant flow diagram. T0, before start 20-week program; T1, halfway 20-week program; T2, after 20-week program; T3, end of 12-month relapse prevention program; RPP, relapse prevention program.

Participants were 3,900 patients with a depressive and/or anxiety disorder as primary diagnoses. Figure 1 shows a flow diagram of all 4,630 referred patients in the period between January 2017 and May 2022. From the 4,630 consecutively referred patients, 730 patients did not start the healthcare program after the first information session and the intake procedures (i.e., phase 1) (i.e., ‘no-go’). The main 20-week healthcare program (i.e., phase 2) was started by 3,900 patients (i.e., ‘go’ – T1). During phase two, 332 patients discontinued the intervention between T0 and T1 and 193 patients discontinued the intervention between T1 and T2 (i.e., total of 525 drop-outs between T0 and T2). Phase 2 was eventually completed by 3,375 patients (i.e., T2). From these 3,375 patients, 2,074 patients were ultimately enrolled in the 12-month RPP (i.e., phase 3). The remaining 1,301 patients decided not to start phase 3 (i.e., ‘no-go’ RPP). During phase three, 1,265 patients discontinued the RPP (i.e., RPP drop-out), leaving 809 patients completing the entire healthcare program (i.e., T0 – T3). Reasons for drop-out during the healthcare program were unfortunately not available in the used database.

Socio-demographic and clinical characteristics of the 3,900 included patients are summarized in Table 1. All participants had a depressive or anxiety disorder as primary diagnosis. Depressive disorders were diagnosed in 61.5% of patients, anxiety disorders in 33.9% of patients and both a depressive and anxiety disorder was diagnosed in 13.3% of patients. Two or more DSM-5 diagnoses were present in 86.9% of patients and three or more DSM-5 diagnoses in 56.1% of patients. Common comorbid DSM-5 diagnoses included: somatic symptom and related disorders (51.7%), trauma-and stressor-related disorders (10.2%), personality disorders (5.7%) and substance-related and addictive disorders (3.6%). See Supplementary Table S3 for a full list of comorbid DSM-5 diagnoses. The duration of complaints was longer than 2 years in 45.3% of the patients. Average age was 45.7 years (SD = 12.3), the majority of patients were working (71.3%) and were married or in a cohabiting relationship (64.7%). Baseline values (i.e., T0) of primary and secondary variables are presented in Tables 2–4. DASS baseline values (i.e., T0) indicate moderate scores for depressive, anxiety and stress symptoms (81). The average baseline value (i.e., T0) for the GSI is more severe compared to established scores for outpatient and inpatient psychiatric patients as reported by Derogatis and Melisaratos (76). Baseline averages for some BSI subscales are markedly higher compared to established psychiatric outpatient norm scores (i.e., somatization and phobic anxiety) and some average baseline BSI subscale scores are lower compared to psychiatric outpatients norm scores (i.e., interpersonal sensitivity, depression, anxiety and hostility) (76). GSI and BSI subscales baseline scores are also more severe compared to established patient norms as reported by De Beurs (101, 102), except for the BSI subscales interpersonal sensitivity and psychoticism which are comparable to the patients norms as reported by De Beurs (101).

**Table 1:** Socio-demographic and clinical characteristics.

Baseline characteristics	*n* = 3,900
Gender, %	
Female	63.4
Male	36.6
Age (years), mean (SD)	45.7 (12.3)
Height (cm), mean (SD)	172.8 (9.5)
Weight (kg), mean (SD)	79.5 (18.5)
BMI, mean (SD)	26.0 (5.4)
Marital status, %	
Single	27.0
Married or cohabiting	64.7
Other	8.2
Level of education^a^, %	
Low	27.6
Middle	40.6
High	31.8
Employment status, %	
Employed	71.3
Unemployed/incapacitated	12.4
Retired	0.9
Housewife/househusband	11.1
Student	4.2
Number of DSM-5 diagnoses, %	
1	13.1
2	30.8
3	29.7
>3	26.4
Primary DSM-5 diagnoses, %	
Depressive disorders	61.5
Anxiety disorders	33.9
Duration of complaints, %	
< 3 months	4.6
3–6 months	15.5
6–12 months	17.5
12–24 months	17.2
> 24 months	45.3

BMI, Body Mass Index; DSM-5, Diagnostic and Statistical Manual of mental disorders, fifth edition; SD, standard deviation; n, number of. ^a^Education level was classified as low (elementary school, high school, lower secondary education), middle (higher secondary education), high (higher professional education, university).

**Table 2:** Means and standard deviations of RAND-36 subscales.

	T0		T1		T2		T3	
	*M*	SD	*M*	SD	*M*	SD	*M*	SD
Emotional role restrictions	15.51	29.91	38.64^a^	40.37	59.17^a,b^	41.06	64.85^a,c^	41.03
General health experience	45.70	18.12	53.27^a^	18.79	60.38^a,b^	20.95	61.95^a^	22.43
Health change	26.33	24.82	52.40^a^	30.18	74.21^a,b^	26.22	73.29^a^	28.81
Mental health	41.53	16.96	56.95^a,*^	16.96	67.07^a,b^	17.97	69.96^a,c^	21.28
Bodily Pain	58.32	24.46	65.97^a^	23.56	71.77^a,b^	23.55	72.94^a^	25.54
Physical functioning	72.67	20.44	77.94^a^	19.15	82.93^a,b^	18.27	82.68^a^	20.48
Physical role restrictions	22.82	34.87	37.10^a^	39.49	52.34^a,b^	41.76	61.98^a,c^	41.74
Social functioning	39.13	22.17	57.66^a^	23.49	69.91^a,b^	23.94	72.50^a,c^	27.09
Vitality	26.42	15.23	42.86^a,*^	18.44	54.89^a,b^	21.25	55.95^a^	23.99
Physical component score	43.72^*^	9.40	45.08^a^	9.27	46.94^a,b^	9.25	47.57^a^	10.26
Mental component score	27.36	9.30	37.21^a^	10.78	44.12^a,b^	11.31	45.69^a,c^	12.56

M, means; SD, standard deviations; T, time. ^a^*p*_adj_ < 0.01; compared to T0, ^b^*p*_adj_ < 0.01; compared to T1, ^c^*p*_adj_ < 0.01; compared to T2, ^*^Normality test significant. For mixed linear models with a main effect of time, post-hoc pairwise comparisons of dependent variable means were carried out between subsequent time points, adjusting for multiple comparison using Bonferroni corrections.

**Table 3:** Means and standard deviations of the brief symptom inventory (BSI) subscales.

	T0		T1		T2		T3	
	*M*	SD	*M*	SD	*M*	SD	*M*	SD
Somatization	1.33	0.79	0.88^a^	0.69	0.64^a,b^	0.65	0.57^a,c^	0.67
Cognitive problems	2.10	0.86	1.58^a^	0.86	1.15^a,b^	0.86	1.01^a,c^	0.90
Interpersonal sensitivity	1.49	0.93	1.17^a^	0.83	0.77^a,b^	0.75	0.64^a,c^	0.72
Depression	1.56	0.86	1.02^a^	0.76	0.66^a,b^	0.71	0.62^a^	0.77
Anxiety	1.61	0.91	1.05^a^	0.73	0.73^a,b^	0.71	0.65^a,c^	0.72
Hostility	0.97	0.74	0.67^a^	0.60	0.43^a,b^	0.49	0.42^a^	0.53
Phobic anxiety	1.08	0.88	0.74^a^	0.70	0.49^a,b^	0.61	0.45^a^	0.64
Paranoid ideation	1.18	0.90	0.94^a^	0.76	0.67^a,b^	0.70	0.56^a,c^	0.69
Psychoticism	1.13	0.72	0.80^a^	0.64	0.52^a,b^	0.57	0.45^a,c^	0.59
Global severity index	1.40	0.61	0.98^a^	0.57	0.68^a,b^	0.55	0.60^a,c^	0.59

M, means; SD, standard deviations; T, time. ^a^*p*_adj_ < 0.01; compared to T0, ^b^*p*_adj_ < 0.01; compared to T1, ^c^*p*_adj_ < 0.01; compared to T2, ^*^Normality test significant. For mixed linear models with a main effect of time, post-hoc pairwise comparisons of dependent variable means were carried out between subsequent time points, adjusting for multiple comparison using Bonferroni corrections.

**Table 4:** Means and standard deviations of DASS scores.

	T0		T1		T2		T3	
	*M*	SD	*M*	SD	*M*	SD	*M*	SD
Anxiety	12.88	8.34	7.93^a^	6.58	5.64^a,b^	6.17	4.98^a,c^	6.28
Depression	18.45	9.79	11.16^a^	8.53	7.39^a,b^	8.03	7.22^a^	8.96
Stress	21.34^*^	8.44	14.77^a^	7.98	10.41^a,b^	7.83	9.60^a,c^	8.40

M, means; SD, standard deviations; T, time. ^a^*p*_adj_ < 0.01; compared to T0, ^b^*p*_adj_ < 0.01; compared to T1, ^c^*p*_adj_ < 0.01; compared to T2, ^*^Normality test significant. For mixed linear models with a main effect of time, post-hoc pairwise comparisons of dependent variable means were carried out between subsequent time points, adjusting for multiple comparison using Bonferroni corrections.

A comparison of baseline characteristics between completers (*n* = 3,375) and drop-outs (*n* = 525) is presented in Supplementary Table S4. Results show significant differences on several baseline variables between drop-outs and completers. Drop-outs report significantly lower scores on the PCS and the RAND-36 subscales general health experience, bodily pain, physical functioning and social functioning. In addition, significantly higher scores among drop-outs are reported on several BSI subscales (i.e., somatization, depression, phobic anxiety, paranoid ideation and psychoticism) and DASS subscales (i.e., anxiety and depression).

### 3.2. Primary outcomes

#### 3.2.1. Health-related quality of life (RAND-36)

MCS and PCS means and standard deviations at each time point are reported in Table 2. The MLM analysis revealed a significant main effect of time on MCS (*F*_2,11,503_ = 3599.0, *p* < 0.05, B = 6.95, 95% CI [6.78, 7.11]) and PCS (*F*_2,11,503_ = 809.2, *p* < 0.05, B = 1.50, 95% CI [1.39, 1.62]). Post-hoc pairwise comparisons of means between subsequent timepoints showed a significant (adjusted for multiple comparisons) increase in MCS between T0 (*M* = 27.36, *SD* = 9.30) and T1 (*M* = 37.21, *SD* = 10.78) (*p_adj_* < 0.01), between T1 (*M* = 37.21, *SD* = 10.78) and T2 (*M* = 44.12, *SD* = 11.31), between T2 (*M* = 44.12, *SD* = 11.31) and T3 (*M* = 45.69, *SD* = 12.56) (*p_adj_* < 0.01). PCS also increased significantly from T0 (*M* = 43.72, *SD* = 9.40) to T1 (*M* = 45.08, *SD* = 9.27) (*p_adj_* < 0.01) and from T1 (*M* = 45.08, *SD* = 9.27) to T2 (*M* = 46.94, *SD* = 9.25) (*p_adj_* < 0.01). The observed increase in PCS between T2 (*M* = 46.94, *SD* = 9.25) and T3 (*M* = 47.57, *SD* = 10.26) was non-significant (*p_adj_* > 0.01). Very Large effect sizes (98) were found for MCS changes from T0 to T2 and T0 to T3. Small effect sizes (97) were found for PCS changes from T0 to T2 and T0 to T3 (Table 5). Changes in MCS score from T0 to T2 (i.e., 16.76) and T0 to T3 (i.e., 18.33) were both clinically significant, based on MCID calculations (i.e., 0.5*SD). However, changes in PCS score from T0 to T2 (i.e., 3.22) and T0 to T3 (i.e., 3.85) were both not clinically significant.

**Table 5:** Cohen’s d effect sizes.

	T0 – T2 *ES*	T0 – T3 *ES*
Primary outcomes		
*RAND-36*		
Mental component summary (MCS)	1.42^+^	1.65^+^
Physical component summary (PCS)	0.33^Ɨ^	0.39^Ɨ^
Emotional role restrictions	1.07^*^	1.36^+^
General health experience	0.68^°^	0.79^°^
Health change	1.65^+^	1.74^+^
Mental health	1.30^+^	1.47^+^
Bodily pain	0.53^°^	0.59^°^
Physical functioning	0.48^Ɨ^	0.48^Ɨ^
Physical role restrictions	0.70^°^	1.00^*^
Social functioning	1.19^*^	1.34^+^
Vitality	1.41^+^	1.46^+^
Secondary outcomes		
*BSI*		
Somatization	−0.90^*^	−1.04^*^
Cognitive problems	−1.01^*^	−1.25^+^
Interpersonal sensitivity	−0.74^°^	−1.03^*^
Depression	−1.02^*^	−1.16^*^
Anxiety	−1.01^*^	−1.18^*^
Hostility	−0.73^°^	−0.84^*^
Phobic anxiety	−0.68^°^	−0.82^*^
Paranoid ideation	−0.56^°^	−0.77^°^
Psychoticism	−0.81^*^	−1.02^*^
Global Severity Index (GSI)	−1.11^*^	−1.33^+^
*DASS*		
Anxiety	−0.90^*^	−1.06^*^
Depression	−1.10^*^	−1.18^*^
Stress	−1.22^+^	−1.37^+^

ES, effect sizes; BSI, Brief Symptom Inventory; RAND-36, Research and Development-36; DASS, Depression Anxiety Stress Scales; MCS, Mental Component Score; PCS, Physical Component Score. Magnitude of ES: ^Ɨ^small ES, ^°^moderate ES, ^*^large ES, ^+^very large ES. Effect size calculation was done based on the independent sample and inequal sample size variant of Cohen’s d as described by Cohen (96). Effect sizes were calculated for changes in dependent variables from T0 (i.e., before start 20-week intervention program) to T2 (i.e., after 20-week intervention program) (left column: T0 – T2 ES) and from T0 (i.e., before start 20-week intervention program) to T3 (i.e., end of 12-month relapse prevention program) (right column: T0 – T3 ES).

RAND-36 subscale means and SD at each time point are also reported in Table 2. MLM analysis yielded a significant effect of time on all RAND-36 subscales: emotional role restrictions (*F*_2,11,503_ = 1747.8, *p* < 0.05, B = 18.00, 95% CI [17.37, 18.64]), general health experience (*F*_2,11,503_ = 1681.4, *p* < 0.05, B = 6.20, 95% CI [5.95, 6.44]), health change (*F*_2,11,503_ = 3107.4, *p* < 0.05, B = 19.02, 95% CI [18.54, 19.51]), general mental health (*F*_2,11,503_ = 3629.0, *p* < 0.05, B = 10.90, 95% CI [10.64, 11.17]), bodily pain (*F*_2,11,503_ = 1091.1, *p* < 0.05, B = 6.05, 95% CI [5.72, 6.37]), physical functioning (*F*_2,11,503_ = 1180.6, *p* < 0.05, B = 4.29, 95% CI [4.06, 4.51]), physical role restrictions (*F*_2,11,503_ = 1213.0, *p* < 0.05, B = 13.37, 95% CI [12.8, 14.0]), social functioning (*F*_2,11,503_ = 2639.5, *p* < 0.05, B = 13.00, 95% CI [12.63, 13.38]) and vitality (*F*_2,11,503_ = 3597.2, *p* < 0.05, B = 11.6, 95% CI [11.3, 11.8]). Post-hoc pairwise comparisons of means between subsequent timepoints and adjusted for multiple comparisons showed a significant (*p_adj_* < 0.01) increase of all RAND-36 subscales between T0 and T1, between T1 and T2 and between T0 and T2. The observed increases in scores on RAND-36 subscales between T2 and T3 were significant for the subscales: emotional role restrictions, general mental health, physical role restrictions and social functioning (*p_adj_* < 0.01). For all other subscales the increases from T2 to T3 were non-significant (*p*_adj_ > 0.01). Scores on two RAND-36 subscales from T2 to T3 decreased, however non-significantly (i.e., health change and physical functioning). Small to very large effect sizes were found for changes in RAND-36 subscales from T1 to T2 and from T0 to T3 (Table 5) (97, 98). Changes on all RAND-36 subscales from T0 to T2 were clinically significant, based on MCID calculations (i.e., 0.5*SD). Also all RAND-36 subscale changes from T0 to T3 were clinically significant, except for the change from T0 to T3 in ‘physical functioning’. See also Supplementary Figure S1 for a visualization of change over time of RAND-36 subscales.

### 3.3. Secondary outcomes

#### 3.3.1. Physical and psychological symptoms (BSI)

GSI means and SD at each time point are reported in Table 3. The MLM analysis revealed a significant main effect of time on GSI (*F*_2,11,526_ = 3436.1, *p* < 0.05, B = − 0.315, 95% CI [−0.323, −0.307]). Post-hoc pairwise comparisons of means between subsequent time points showed a significant (adjusted for multiple comparisons) decrease in GSI between T0 (*M* = 1.40, *SD* = 0.61) and T1 (*M* = 0.98, *SD* = 0.57) (*p_adj_* < 0.01), between T1 (*M* = 0.98, *SD* = 0.57) and T2 (*M* = 0.68, *SD* = 0.55), between T2 (*M* = 0.68, *SD* = 0.55) and T3 (*M* = 0.60, *SD* = 0.59) (*p_adj_* < 0.01). Large effect sizes (97) were found for GSI changes from T0 to T2 and very large effect sizes (98) for GSI changes from T0 to T3 (Table 5).

BSI subscale means and SD at each time point are also reported in Table 3. MLM analysis yielded a significant effect of time on all BSI subscales: somatization (*F*_2,11,526_ = 2324.5, *p* < 0.05, B = − 0.304, 95% CI [− 0.313, − 0.294]), cognitive problems (*F*_2,11,526_ = 2787.2, *p* < 0.05, B = − 0.407, 95% CI [− 0.419, − 0.396]), interpersonal sensitivity (*F*_2,11,526_ = 1902.0, *p* < 0.05, B = − 0.309, 95% CI [− 0.320, − 0.298]), depression (*F*_2,11,526_ = 2661.8, *p* < 0.05, B = − 0.386, 95% CI [− 0.398, − 0.375]), anxiety (*F*_2,11,526_ = 2574.3, *p* < 0.05, B = − 0.388, 95% CI [− 0.400, − 0.377]), hostility (*F*_2,11,526_ = 1609.1, *p* < 0.05, B = − 0.227, 95% CI [− 0.237, − 0.218]), phobic anxiety (*F*_2,11,526_ = 1691.9, *p* < 0.05, B = − 0.258, 95% CI [− 0.268, − 0.247]), paranoid ideation (*F*_2,11,526_ = 1388.2, *p* < 0.05, B = − 0.221, 95% CI [− 0.231, − 0.211]) and psychoticism (*F*_2,11,526_ = 1933.9, *p* < 0.05, B = − 0.266, 95% CI [− 0.276, − 0.257]). Post-hoc pairwise comparisons of means between subsequent time points and adjusted for multiple comparisons showed a significant (*p_adj_* < 0.01) decrease of all BSI subscales between T0 and T1, between T1 and T2 and between T0 and T2. The observed decreases in scores on BSI subscales between T2 and T3 were significant (*p_adj_* < 0.01) for the subscales: somatization, cognitive problems, interpersonal sensitivity, anxiety, paranoid ideation and psychoticism. Decreases in BSI subscale scores from T2 to T3 were non-significant for the subscales: depression, hostility and phobic anxiety (*p_adj_* > 0.01). Medium to large effect sizes (97) were found for changes for all BSI subscales between T0 and T2 (Table 5). In addition, medium to very large effect sizes (97, 98) were found for BSI subscale changes between T0 and T3 (Table 5). See also Supplementary Figure S2 for a visualization of change over time of BSI subscales.

#### 3.3.2. Symptoms of depression, anxiety, and stress (DASS)

DASS means and SD at each time point are reported in Table 4. MLM analysis yielded a significant effect of time on all DASS subscales: anxiety (*F*_2,11,481_ = 2228.5, *p* < 0.05, B = − 3.146, 95% CI [− 3.249, − 3.043]), depression (*F*_2,11,481_ = 2765.4, *p* < 0.05, B = − 4.638, 95% CI [− 4.771, − 4.505]) and stress (*F*_2,11,481_ = 3123.1, *p* < 0.05, B = − 4.535, 95% CI [− 4.654, − 4.415]). Post-hoc pairwise comparisons of means between subsequent time points and adjusted for multiple comparisons showed significant (*p_adj_* < 0.01) decreases of all DASS subscales scores from T0 to T1, from T1 to T2 and from T0 to T2. The observed decreases in DASS subscale scores from T2 to T3 were significant (*p_adj_* < 0.01) for the subscales anxiety and stress and non-significant for the subscale depression (*p_adj_* > 0.01). Moreover, 57% of the patients showed a clinical response (i.e., ≥ 50% reduction of DASS depression score) in depressive symptoms at T2 and 66% at T3. For anxiety symptoms, 45% of patients showed a clinical response (i.e., ≥ 50% reduction in DASS anxiety score) at T2 and 53% at T3. Remission rates for depressive symptoms were 43% at T2 and 63% at T3 (i.e., DASS depression score ≤ 9) (81). Remission rates for anxiety symptoms were 50% at T2 and 67% at T3 (i.e., DASS anxiety score ≤ 7). In addition, large to very large effect sizes (97, 98) were found for DASS subscale score changes from T0 to T2 and from T0 to T3 (Table 5). See also Supplementary Figure S3 for a visualization of change over time of DASS subscales.

## 4. Discussion

The purpose of this study was to evaluate the effectiveness of an outpatient secondary care interdisciplinary multimodal integrative healthcare program for patients with depressive and/or anxiety disorders. The results seem to indicate that an interdisciplinary multimodal healthcare program and subsequent RPP contributes to the recovery of patients with depressive and/or anxiety disorders. Results revealed significant improvements from T0 to T2 (i.e., phase 2) for the primary variable HRQoL (i.e., RAND-36) and for all secondary variables, including: current psychological and physical symptoms (i.e., BSI) and symptoms of depression, anxiety and stress (i.e., DASS). Moreover, improvements attained during phase 2 in primary and secondary variables were not only maintained during phase 3 (i.e., T2 – T3), but significant improvements were observed for the majority of variables. In contrast to our expectations, further significant improvements during phase 3 (i.e., T2 – T3) were mainly observed for secondary variables (i.e., BSI and DASS) and to a lesser extent for the primary variable (i.e., HRQoL measured with RAND-36). Effect sizes for the primary variable HRQoL ranged from small for improvements in physical HRQoL (i.e., PCS) to very large for improvements in mental HRQOL (i.e., MCS). Effect sizes for RAND-36 subscales ranged from small to very large. For secondary variables, effect sizes ranged from medium to very large for BSI subscales and from large to very large for DASS subscales. Remission rates were 63% for depressive symptoms (i.e., DASS depression score ≤ 9) and 67% for anxiety symptoms (i.e., DASS anxiety score ≤ 7) at T3.

The current study extents on previous research that reported on the effectiveness of multimodal interventions in (comorbid) depressive and anxiety disorders (31, 40–45). Reported effect sizes for the reduction in depressive and anxiety symptoms from baseline to post-treatment were comparable across most of these studies, including the current study, mostly ranging from large to very large (31, 41, 43). Since high rates of relapse/recurrence have been reported in depressive and anxiety disorders, it is important to consider long-term stability of intervention results. Relapse rates of 10 to 49% within one year after treatment have been reported for depressive disorders (59, 103, 104). For anxiety disorders, relapse rates of 14% are reported (105, 106). Continuation-phase interventions can be delivered to reduce relapse rates and maximize the long-term benefits of therapy (59). Even after continuation-phase interventions, relapse rates of 29% are reported for depressive disorders (59). Among the aforementioned multimodal interventions, only two studies evaluated the long-term stability of their results (40, 41). Both studies reported that intervention results were maintained from post-treatment to follow-up, despite the fact that depressive and anxiety symptoms started to increase again once treatment was completed (40, 41). It is however important to note that Herzog et al. (41) provided a continuation-phase intervention during the follow-up period (i.e., support groups or 4-week refresher course), whereas Hauksson et al. (40) did not provide a continuation-phase intervention during follow-up. The current study provided a 12-month blended RPP after the end of phase 2 (20-week main intervention: T0 – T2). Surprisingly, the results from the current study seem to indicate that after phase 2 (i.e., T0 – T2), psychological and physical symptoms (i.e., BSI) and symptoms of depression, anxiety and stress (i.e., DASS) continue to decrease until the end of phase 3 (i.e., end of RPP). Remission rates for depressive symptoms were 43% after phase 2 (T0 – T2) and 63% at the end of phase 3 (i.e., end of RPP) (i.e., DASS depression score ≤ 9) (81). Remission rates for anxiety symptoms were 50% after phase 2 and 67% at the end of phase 3 (i.e., end of RPP) (i.e., DASS anxiety score ≤ 7). Moreover, quality of life measures also continued to improve from T2 to T3 (i.e., phase 3), but to a lesser extent compared to the secondary variables. These results add to the current available evidence for the feasibility of blended interventions in relapse prevention as a possible continuation-phase intervention (107).

When comparing the aforementioned multimodal interventions, several differences among these studies can be discussed. First, all of the aforementioned studies provided inpatient multimodal interventions (31, 38, 40, 41, 43) in contrast to the outpatient multimodal intervention evaluated in the current study. Second, the majority of these studies, including the current study, described a ‘time-limited’ intervention program (38, 40, 41) with a fixed intervention length, instead of an open-ended intervention length (31, 43) where patient and therapist decide together when to end therapy. Third, treatment length and frequency were considerably heterogeneous across the aforementioned multimodal interventions (31, 38, 40, 41, 43). Intervention length ranged from 5 weeks (31) to 30 weeks (43) in the open-ended interventions and from 6 weeks (40) to 20 weeks (i.e., current study) in the ‘time-limited’ interventions. Treatment frequency ranged from two full days a week (41) to five full days a week (38, 40). Despite the observed heterogeneity in health care setting (i.e., inpatient vs. outpatient), treatment length and treatment frequency across the aforementioned studies, treatment outcomes are comparable. This is important to consider when taking healthcare costs into consideration, because inpatient, high intensive, open-ended interventions are usually more expensive in comparison to outpatient time-limited interventions.

In addition to treatment dose, considerably heterogeneity is also observed in treatment content among the previously mentioned studies (31, 40, 41, 43). Most of these studies mention six to eight treatment components, including: (1) psychiatric, medical and/or nursing care, (2) pharmacotherapy and/or medication management, (3) lifestyle interventions (with focus on healthy nutrition, sleep, physical activity and relaxation), (4) psychotherapy (individual and/or group), (5) exercise therapy, (6) psycho-education, (7) systems therapy, (8) social/recreational activities, (9) art therapy, (10) music therapy, (11) social competence training, (12) CBT and (13) electro convulsive therapy. Also the number of involved healthcare professionals is variable, ranging from three professionals (i.e., current study) to six professionals (40). Despite the heterogeneity with regard to individual treatment components and involved healthcare personnel, the results are nevertheless comparable across the aforementioned multimodal interventions. A possible interpretation for the comparable results across these studies could be that the benefits of these multimodal interventions is not necessarily explained by specific treatment components, but more likely due to the fact that these multimodal interventions apply the same underlying treatment principle across a wider range of diagnosed mental disorders, while individualizing treatment components for each patient. These results could suggest that multimodal interventions are best delivered within a transdiagnostic framework. In fact, a growing body of evidence is supporting the use of transdiagnostic interventions for individuals experiencing depression and anxiety symptoms (16, 22, 24).

A final important aspect of the healthcare program evaluated in the current study is that the exercise therapy was delivered by physical therapist, in contrast to most other multimodal interventions where different healthcare professionals usually deliver the exercise interventions. Since physical therapist are movement/somatic experts, they are prime candidates to address the bodily symptoms of depressive and anxiety disorders. To date, a growing body of studies support the use of exercise therapy for depressive (108–110) and anxiety disorders (111–114), both as mono-intervention and as add-on for CBT (115, 116). However, no evidence exists for the added value of exercise interventions within multimodal interventions for depressive and anxiety disorders. Furthermore, it is unknown whether exercise interventions delivered by physical therapists are superior compared to exercise interventions delivered by other healthcare professionals (108). Only one promising RCT by Bratberg et al. (117) compared physical therapy treatment with standard psychological/psychiatric treatment for patients experiencing mixed anxiety and depression. Results showed superior results for reducing depressive symptoms and improving quality of life in favor of physical therapy at short-term but no differences between both therapies were found at follow-up (117). Future studies should further investigate the added value of physical therapists delivering exercise interventions in depressive and anxiety disorders.

There are a few limitations concerning this study. First, the current study used a descriptive study design (i.e., case series). Internal validity of case series is usually very low, due to the absence of a comparator group. Therefore, it is not possible to conclude with certainty whether the observed effects are due to the healthcare program or other factors (i.e., time effects, etc.). Second, it is not possible to discern whether results reported at the end of phase 3 (i.e., end RPP) are in fact sustained effects of the initial intervention (i.e., phase 2) or only due to the RPP (i.e., phase 3). Third, it was not possible to provide conclusive evidence for the long-term stability of treatment results after the two active intervention phases (i.e., main 20-week intervention and RPP), as the current study did not include a follow-up period after completion of the 12-month RPP (i.e., phase 3). Fourth, reasons for drop-out were not registered in the used database, which is unfortunate since the drop-out rates in the current study are substantial. It is however important to mention that premature discontinuation of phase 2 (i.e., T0 – T2) due to the achievement of treatment goals in an earlier stadium (i.e., before end of phase 2) was also considered a drop-out in the current study. Finally, it is not possible to generalize the results beyond the used study sample despite the large sample size. It is unclear to what extent results can be generalized to patients with comorbid depression and anxiety, since only 13% of patients in the current study were diagnosed with both a depressive and anxiety disorder. In addition, case series are usually also vulnerable to selection bias, which makes generalization more difficult. Strengths of the current study are the use of a realistic study sample of participants with comorbid DSM-5 diagnoses, instead of single DSM-5 diagnoses. The ulilization of real-world data in an effectiveness study and the incorporation of a continuation-phase intervention (i.e., RPP) are other strengths.

The results of the current study are of added value to the existing evidence base consisting of mainly efficacy studies (38, 118–124) for the use of these interdisciplinary multimodal interventions for patients suffering from depression and/or anxiety disorders. Moreover, Smeets (125) specifically pleaded for the use of alternative research designs, such as Single Case Replicated Designs (SCED) and observational studies of routinely collected data, in order to add to the existing evidence base mainly consisting of randomized controlled trials, since the clinical applicability of these alternative research designs might be higher. Therefore, the current descriptive study, using routinely collected data of a large patient group, might be of added value to policy makers and/or health insurers in the weighting process whether or not to reimburse interdisciplinary multimodal interventions. This is important since reimbursement and funding of these interdisciplinary multimodal interventions in different patient groups has been under pressure in recent years (125, 126).

Future research should further investigate the use of (blended) continuation-phase interventions in patients with depression and/or anxiety disorders. In addition, more studies are warranted to investigate the long-term stability of treatment results after interdisciplinary multimodal intervention for this patient group. Another important aspect that needs further attention is the added value of physical therapists delivering exercise interventions as compared to other healthcare professionals delivering these interventions for patients with depression and/or anxiety disorders. Finally, also alternative research designs, such as SCED (127), can be used to better capture the idiosyncratic nature of treatment in mental health disorders and to investigate the added value of individual treatment components (i.e., CBT, lifestyle interventions, exercise therapy, etc.) within interdisciplinary multimodal interventions.

In conclusion, the results of the current study are of added value to the existing evidence base for the use of interdisciplinary multimodal interventions for patients with (comorbid) depressive and/or anxiety disorders. In addition, results provide preliminary evidence for the value of using a blended relapse prevention program as continuation-phase intervention.

## Data availability statement

The raw data supporting the conclusions of this article will be made available by the authors, without undue reservation.

## Ethics statement

The current study was reviewed and approved by Medical Ethics Review Committee of Maastricht University Medical Center (MUMC+), P. Debyelaan 25, Postbus 5800, 6202 AZ Maastricht, Attn: METC azM/UM. The patients provided their written informed consent to participate in this study.

## Author contributions

JW: conceptualization, methodology, writing – original draft, and project administration. NG: writing—review and editing. GH: writing—review and editing. MP: methodology, formal analysis, data curation, and visualization. MG: methodology, formal analysis, data curation, and visualization. JO: writing—review and editing. JJ: conceptualization and supervision. All authors contributed to the article and approved the submitted version.

## Conflict of interest

JW, NG, GH, MP, MG, and JJ are employed by Intergrin Academy. Intergrin Academy evaluates and improves specialized secondary care formats such as offered at ‘PHI’. Intergrin Academy and ‘PHI’ are part of the same overarching organization: Integrative Interventions Group. In addition, JW was a former employee of ‘Het Rughuis’, a mental healthcare facility also part of Integrative Interventions Group.

## Publisher’s note

All claims expressed in this article are solely those of the authors and do not necessarily represent those of their affiliated organizations, or those of the publisher, the editors and the reviewers. Any product that may be evaluated in this article, or claim that may be made by its manufacturer, is not guaranteed or endorsed by the publisher.

## Glossary

BSI: Brief Symptom Inventory
CBT: Cognitive Behavioral Therapy
DASS: Depression Anxiety Stress Scale
DSM: Diagnostic and Statistical Manual of Mental Disorders
GSI: Global Severity Index
HRQoL: Health Related Quality of Life
MCID: Minimal Clinically Important Difference
MCS: Mental Component Summary
MDD: Major Depressive Disorder
MLM: Mixed Linear Model
NICE: National Institute of Health and Care Excellence
PCS: Physical Component Summary
PHI: Premium Healthcare Interventions
RAND-36: Research and Development 36
RPP: Relapse Prevention Program
SCED: Single Case Replicated Designs
UK: United Kingdom
WMO: Wet Medisch-wetenschappelijk Onderzoek met mensen
