# Supplementary material for: An interdisciplinary multimodal integrative healthcare program for depressive and anxiety disorders
Source: Front Psychiatry. 2023 Jun 23;14:1113356. doi: 10.3389/fpsyt.2023.1113356 (PMC10326275; doi:10.3389/fpsyt.2023.1113356)
Supplement: Supplementary file 1 [file Data_Sheet_1.DOCX]

Supplementary Material

An interdisciplinary multimodal integrative healthcare program for depressive- and anxiety disorders

Jaap Wijnen, Nicole Louise Gordon, Geert van ’t Hullenaar, Marc Lucas Pont, Marciano Wilhelmina Henricus Geijselaers, Jessica Van Oosterwijck and Jeroen de Jong

# Supplementary Figures and Tables

## Supplementary Figures
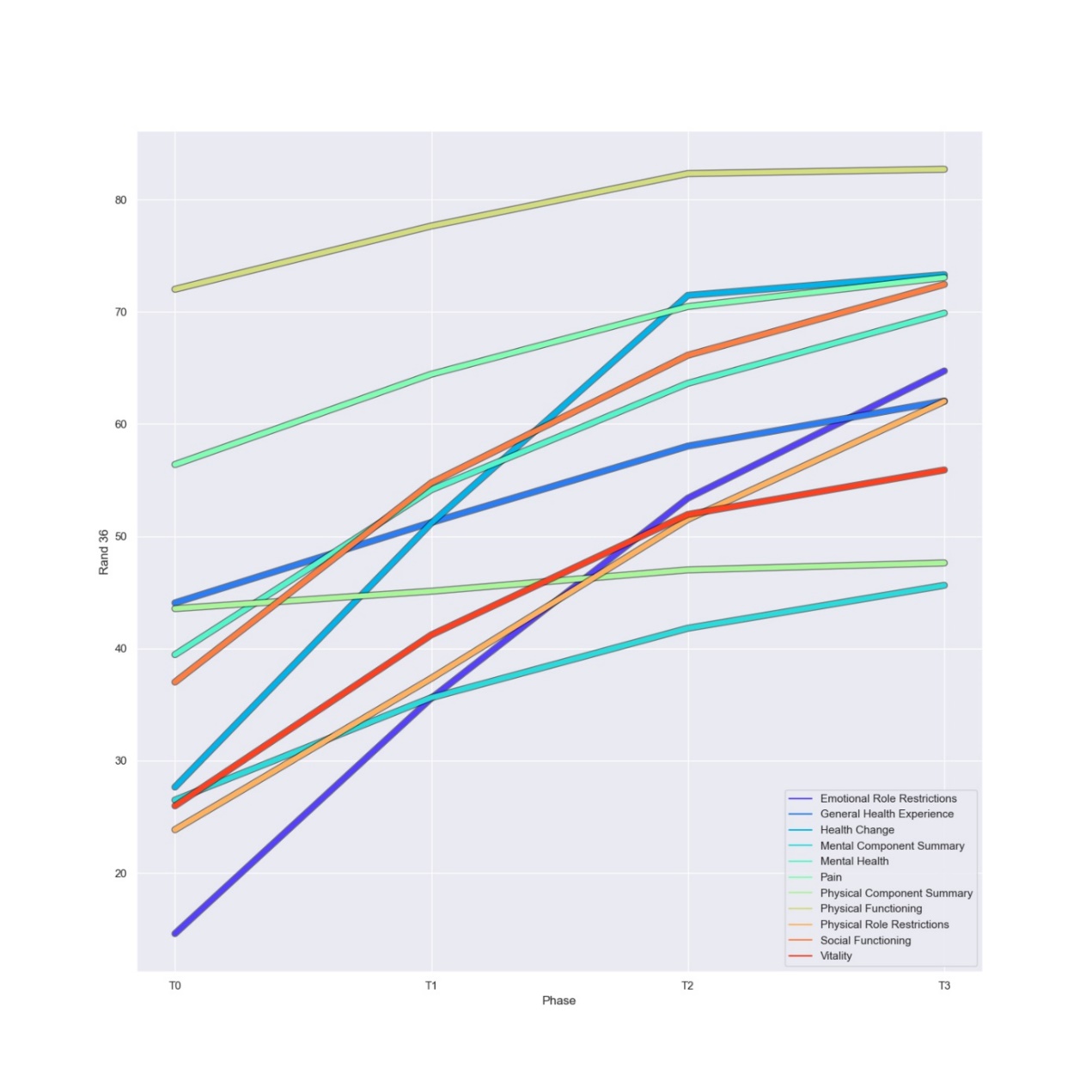


**Supplementary Figure 1.** RAND-36 subscale averages over time.


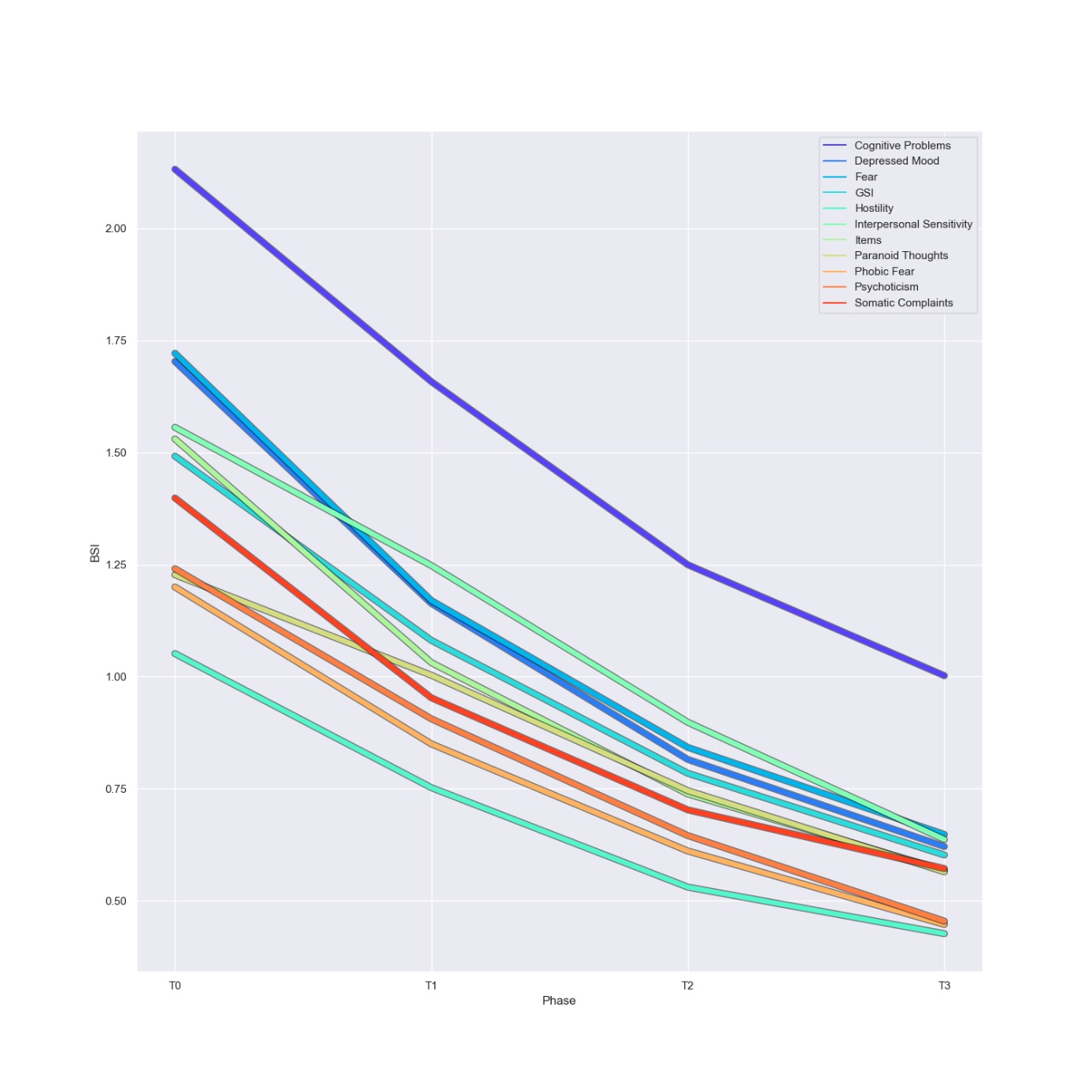


**Supplementary Figure 2.** Brief Symptom Inventory (BSI) subscale averages over time


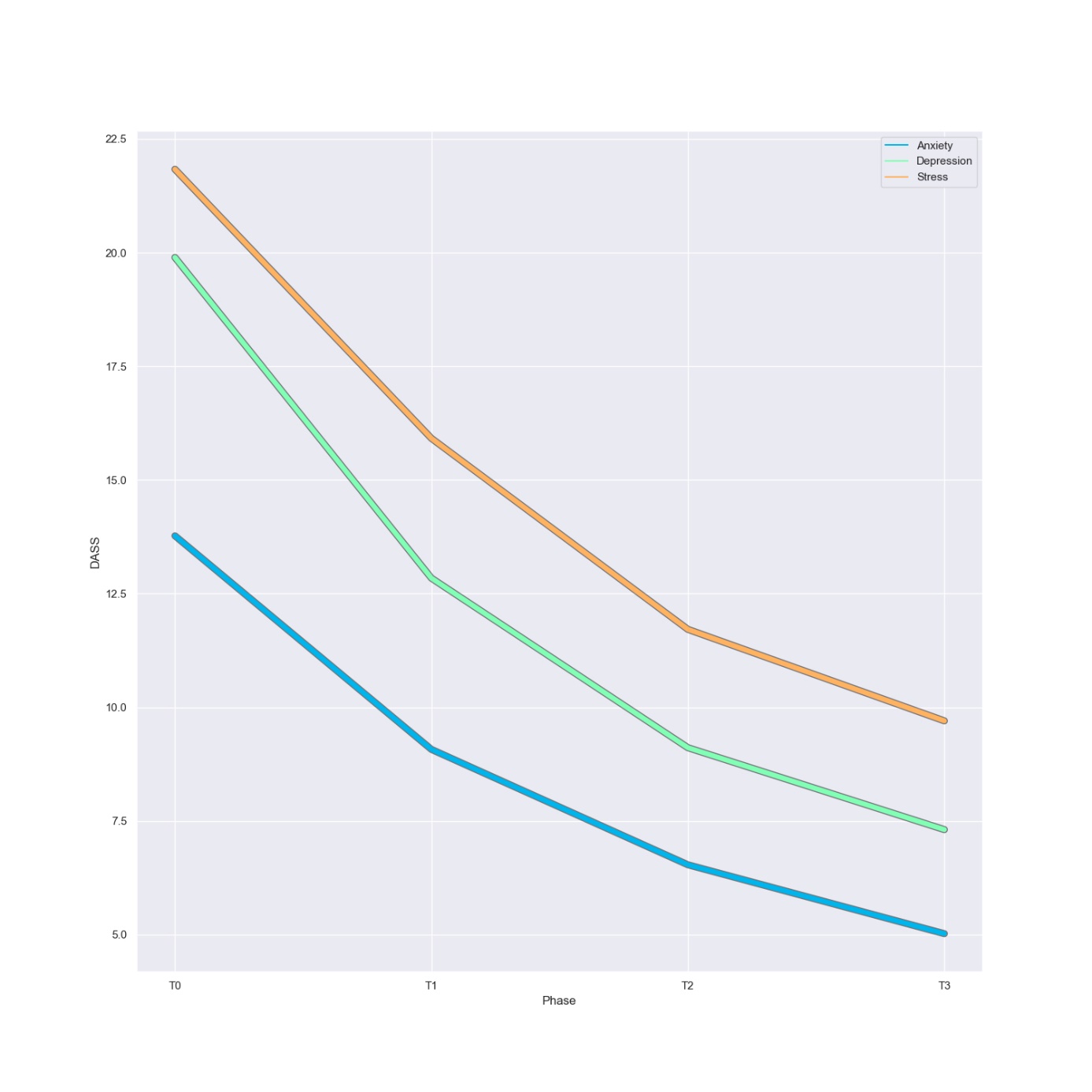


**Supplementary Figure 3.** DASS subscale averages over time

## Supplementary Tables

**Supplementary table 1. Provider information**

| Health care professionals | Number of providers^*^ | Mean (SD) patients per provider^**^ |
| --- | --- | --- |
| Psychiatrists | 7 | 595 (791) |
| Coordinating practitioners | 32 | 195 (214) |
| Physical therapists | 30 | 212 (216) |
| Psychologists | 282 | 103 (92) |
| SD = standard deviation  Notes: ^*^Number of involved healthcare professionals in complete sample (n = 3900), ^**^Mean number (SD) of patients per provider over the period between January 2017 and May 2022  **Supplementary Table 2. Intervention modules and duration**   \| Phase \| Duration \| Overall theme and components \| \| --- \| --- \| --- \| \| Intake (phase 1) \| 600 minutes \| Psychosomatics, lifestyle, cognition and behavior and somatic factors \| \|  \|  \|  \| \| Main 20-week program (phase 2) \| \| \| \| Module 1 \| 690 minutes \| Introduction, goal formulation, case conceptualization and (psycho)-education \| \| Module 2 \| 660 minutes \| Exploration/recognition of maladaptive behavioral patterns and thoughts, emotion regulation, body awareness, mentalization and behavioral activation \| \| Module 3 \| 930 minutes \| Focus on personal context of patient, behavioral experiments and exposure \| \| Module 4 \| 630 minutes \| Strengthening capacity to autonomously cope/manage with physical, emotional and social challenges, encouragement for behavioral change and existential themes \| \| Module 5 \| 960 minutes \| Future goals, sustainability of treatment results and relapse prevention \| \|  \|  \|  \| \| Twelve month Relapse Prevention Program (phase 3) \| \| \| \| RPP \| 600 minutes \| Sustainability of treatment results, encouragement of self-management, behavioral change maintenance, repetition of main themes covered during the 20-week main intervention, discussing pitfalls/risks for relapse \|   RPP = relapse prevention program | | |

**Supplementary Table 3. Comorbid DSM-5 diagnoses**

| DSM-5 diagnosis | Count^*^ | %^**^ |
| --- | --- | --- |
|  |  |  |
| Somatic symptom and related disorders | 2016 | 51.7 |
| Trauma- and stressor-related disorders | 396 | 10.2 |
| Personality disorders | 221 | 5.7 |
| Substance-related and addictive disorders | 140 | 3.6 |
| Neurodevelopmental disorders | 102 | 2.6 |
| Obsessive compulsive and related disorders | 68 | 1.7 |
| Feeding and eating disorders | 39 | 1.0 |
| Sleep-wake disorders | 25 | 0.6 |
| Sexual dysfunctions | 6 | 0.2 |
| Disruptive, impulse-control and conduct disorders | 6 | 0.2 |
| Other mental disorders | 5 | 0.1 |
| Neuro-cognitive disorders | 4 | 0.1 |
| Dissociative disorders | 4 | 0.1 |
| Medication-induced movement disorders and other adverse effect of medication | 1 | 0.0 |
|  |  |  |
| Notes: ^*^Number of times DSM-5 diagnoses were assigned within the complete sample; ^**^Proportion of participants within the complete sample (*n* = 3900) receiving a particular DSM-5 diagnosis in addition to depressive- and/or anxiety disorder. | | |

**Supplementary Table 4. Comparison drop-outs and completers**

| Baseline characteristics | Drop-outs  (n = 525) | | Completers  (n = 3375) | | | p-value |  |
| --- | --- | --- | --- | --- | --- | --- | --- |
|  | *M* | *SD* | *M* |  | *SD* | | |
| Number of DSM-5 diagnoses | 3.04 | (1.49) | 2.83 | | (1.30) | 0.002^*^ |  |
|  |  |  |  | |  |  |  |
| Primary outcomes |  |  |  | |  |  |  |
| RAND-36 |  |  |  | |  |  |  |
| MCS | 26.63 | (9.41) | 26.47 | | (9.17) | 0.722 |  |
| PCS | 42.05 | (10.45) | 43.79 | | (9.78) | 0.000^*^ |  |
| Emotional role restrictions | 15.24 | (28.91) | 14.49 | | (28.57) | 0.580 |  |
| General health experience | 40.46 | (18.08) | 44.62 | | (18.31) | 0.000^*^ |  |
| Health change | 29.33 | (26.01) | 27.39 | | (25.84) | 0.111 |  |
| Mental health | 38.13 | (18.06) | 39.65 | | (17.00) | 0.071 |  |
| Pain | 51.83 | (27.21) | 57.09 | | (25.22) | 0.000^*^ |  |
| Physical functioning | 68.92 | (24.60) | 72.48 | | (20.92) | 0.001^*^ |  |
| Physical role restrictions | 23.62 | (35.11) | 23.89 | | (36.09) | 0.870 |  |
| Social functioning | 34.83 | (24.68) | 37.34 | | (22.90) | 0.029^*^ |  |
| Vitality | 27.14 | (15.24) | 25.79 | | (15.11) | 0.059 |  |
|  |  |  |  | |  |  |  |
| Secondary outcomes |  |  |  | |  |  |  |
| BSI |  |  |  | |  |  |  |
| Global Severity Index | 1.59 | (0.70) | 1.48 | | (0.64) | 0.000^*^ |  |
| Somatization | 1.51 | (0.87) | 1.38 | | (0.82) | 0.002^*^ |  |
| Cognitive problems | 2.10 | (0.91) | 2.14 | | (0.86) | 0.455 |  |
| Interpersonal sensitivity | 1.60 | (1.04) | 1.55 | | (0.94) | 0.250 |  |
| Depression | 1.87 | (0.96) | 1.68 | | (0.88) | 0.000^*^ |  |
| Anxiety | 1.79 | (1.01) | 1.71 | | (0.94) | 0.116 |  |
| Hostility | 1.10 | (0.87) | 1.04 | | (0.80) | 0.160 |  |
| Phobic anxiety | 1.29 | (0.98) | 1.19 | | (0.94) | 0.017^*^ |  |
| Paranoid ideation | 1.35 | (0.99) | 1.21 | | (0.91) | 0.002^*^ |  |
| Psychoticism | 1.36 | (0.84) | 1.22 | | (0.76) | 0.000^*^ |  |
| DASS |  |  |  | |  |  |  |
| Anxiety | 15.31 | (9.41) | 13.53 | | (8.62) | 0.000^*^ |  |
| Depression | 21.49 | (10.63) | 19.65 | | (9.95) | 0.000^*^ |  |
| Stress | 21.53 | (8.72) | 21.88 | | (8.41) | 0.390 |  |

M = means; SD = standard deviations (SD), MCS = mental component summary, PCS = physical component summary, BSI = Brief Symptom Inventory; RAND-36 = Research and Development-36; DASS = Depression Anxiety Stress Scales

Notes: ^*^*p* < 0.05 on independent samples t-test

# Supplementary Data

## Archive link Statsmodels website

<https://web.archive.org/web/20230419111706/https://www.statsmodels.org/stable/mixed_linear.html>

## Python code mixed linear models

import pandas as pd

import numpy as np

# This method is pulled from a notebook style analysis hence the use of global variables.

# The global variable scores is a dict[str, pd.DataFrame]

def create_mlm(survey_name):

survey_scores = scores[survey_name]

cell = [1, 1]

tab_title = f'Linear Mixed Model {survey_name}'

for sub_score in sorted(survey_scores['Score'].unique()):

sub_survey = survey_scores[survey_scores['Score'] == sub_score]

contrasted = ['C(Phase)', 'phase_num'] # , 'phase_day'] # , '(Phase : DSM5_Count)']

column = cell[1]

row_diff = 0

for item in contrasted:

# if (test_counter == 0): # & (sub_score == 'Mental Health'):

md = smf.mixedlm(f'Result ~ {item}', sub_survey,

groups=sub_survey['UUID'], exog_re=None)

mdf = md.fit(reml=False) # pylint: disable=E1123

# logger.debug(f'Model fit for {sub_score} contrasted on {item}')

# create_mlm_images(mdf, sub_survey)

row = cell[0]

column_diff = 0

for index, table in enumerate(mdf.summary().tables):

if column_diff == 0:

xlsx.df_to_wb_tab(table, wb, tab_title,

f'{sub_score} ~ {item}', start=(row, column))

else:

xlsx.df_to_wb_tab(table, wb, tab_title, start=(row, column))

row += (len(table.index) + 2)

column_diff = max(column_diff, len(table.columns) + 2)

row_diff = row

A = np.identity(len(mdf.params))

A = A[1:, :]

f_test = mdf.f_test(A)

df_f_test = pd.DataFrame([[f_test.fvalue, f_test.pvalue, f_test.df_num, f_test.df_denom]], columns=[

'F value', 'p value', 'df1', 'df2'])

xlsx.df_to_wb_tab(df_f_test, wb, tab_title, start=(row_diff, column))

row_diff += len(df_f_test) + 2

column += column_diff

cell[0] = row_diff + 2

## Python code normality testing and pairwise comparisons

import itertools

from typing import List, Tuple

import numpy as np

import pandas as pd

import scipy as sp

def calculate_survey_stats(scores: pd.DataFrame, phases: List[str], N: int, alpha=0.05):

# H(0): The results come from a normal distribution (p<=α with α=0.05).

def normaltest(series, alpha=0.05):

p = (1 if len(series) <= 8 else sp.stats.normaltest(series)[1])

a = alpha

return {'p': p, 'a': a}

survey_stats = (scores

.groupby(['Phase', 'Score'])

.agg(mean=('Result', # type:ignore

lambda r: np.mean(r.astype(np.float64))),

std=('Result',

lambda r: np.std(r.astype(np.float64))),

normality=('Result', lambda r: normaltest(r, alpha=alpha)))

.transpose()

[phases])

delta = (survey_stats.loc['mean', :].astype(np.float64) /

survey_stats.loc['mean', 'Intake'].astype(np.float64))

delta = delta.loc[[p for p in phases if p != 'Intake'], :]

delta = delta.rename({p: f'Intake=>{p}' for p in phases}, level=0, axis=0)

delta.name = 'delta'

delta = (delta.abs() - 1 * np.sign(delta))

survey_stats = survey_stats.append(delta, sort=False)

# H(0): Improvements are stat. significant w.r.t. predecessors (p<=α with α=0.05)

def H0(score, pre_phase, post_phase, alpha=0.05):

'''Use a T-Test if assumed normally distributed, otherwise use Wilcoxon.'''

a = alpha

normality = survey_stats.loc['normality', (post_phase, score)]

post_phase = scores[(scores['Score'] == score)

& (scores['Phase'] == post_phase)

].drop_duplicates(['UUID'], 'last')

pre_phase = scores[(scores['Score'] == score)

& (scores['Phase'] == pre_phase)

].drop_duplicates(['UUID'], 'last')

pre_phase = pre_phase.merge(post_phase['UUID'], how='inner')

post_phase = post_phase.merge(pre_phase['UUID'], how='inner')

pre_phase = pre_phase['Result']

post_phase = post_phase['Result']

if normality['p'] <= normality['a']:

p = sp.stats.ttest_rel(pre_phase, post_phase)[1]

elif (pre_phase == post_phase).all():

p = np.nan

else:

p = sp.stats.wilcoxon(pre_phase, post_phase)[1]

return {'p': p, 'a': a}

def statistical_significance(series: pd.Series, alpha=0.05):

d = {survey_stats.columns.names[i]: series.name[i] # type:ignore

for i in range(len(series.name))} # type:ignore

pre_phase = d['Phase']

score = d['Score']

post_phases = []

if pre_phase == 'Intake':

post_phases = ['IE', 'FE', 'SCP 12']

elif pre_phase == 'IE':

post_phases = ['FE']

elif pre_phase == 'FE':

post_phases = ['SCP 6', 'SCP 12']

else:

return [np.nan]

post_phases = [p for p in post_phases

if p in survey_stats.columns.get_level_values(0)]

if len(post_phases) <= 0:

return [np.nan]

# Pandas dislikes dictionaries, so let it unpack a list of 1 item

return [{p: H0(score, pre_phase, p, alpha) for p in post_phases}]

survey_stats = survey_stats.append(survey_stats.apply(

lambda r: statistical_significance(r, alpha)))

survey_stats = survey_stats.rename({0: 'stat. sig.'})

survey_stats = survey_stats.transpose().unstack('Phase').swaplevel(axis=1).dropna(axis=1)

survey_stats.columns = survey_stats.columns.rename('Measurement', level=1)

survey_stats[('Population', 'N')] = scores.groupby('Score')['UUID'].nunique()

survey_stats[('Population', '%')] = survey_stats[('Population', 'N')] / N

survey_stats.loc[:, (slice(None), ['mean', 'std', 'delta', 'N', '%'])] = (

survey_stats.loc[:, (slice(None), ['mean', 'std', 'delta', 'N', '%'])]

.astype(np.float64))

column_order = (

[(y, x) for x, y in itertools.product(['mean', 'std', 'normality'], phases)]

+ [(y, x) for x, y in itertools.product(['delta'],

[f'Intake=>{p}' for p in phases if p != 'Intake'])]

+ [(y, x) for x, y in itertools.product(['stat. sig.'], phases)]

)

column_order = [c for c in column_order if c in survey_stats.columns]

return survey_stats[column_order]

def get_survey_stats(scores: pd.DataFrame, phases, alpha=0.05) -> Tuple[pd.DataFrame, pd.DataFrame]:

scores = scores[scores['Phase'].isin(phases)]

uuids = scores.groupby('UUID').nunique()['Phase']

uuids = uuids[uuids >= len(phases)].index.tolist()

scores = scores[scores['UUID'].isin(uuids)]

N = scores['UUID'].nunique()

raw_stats = calculate_survey_stats(scores, phases, N, alpha=alpha)

phase_int = {phases[i]: i + 1 for i in range(len(phases))}

survey_p_vals = raw_stats.loc[:,

(slice(None), ['normality', 'stat. sig.'])]

survey_normality = (survey_p_vals.loc[:, (slice(None), 'normality')]

.droplevel('Measurement', axis=1).stack()

.apply(pd.Series)

.apply(lambda r: r['p'] if r['p'] > r['a'] else np.nan,

axis=1)

.unstack('Phase'))

survey_stat_sig = (survey_p_vals.loc[:, (slice(None), ['stat. sig.'])]

.droplevel('Measurement', axis=1).stack()

.apply(pd.Series)

.rename_axis(columns='Post').stack().apply(pd.Series)

.apply(lambda r: r['p'] if r['p'] > r['a'] else np.nan,

axis=1)

.unstack(['Phase', 'Post']))

norm_s = (lambda p: f"$n_{phase_int[p]}$: {p} is not normally distributed")

normality_footnotes = (survey_normality.any().reset_index()

.apply((lambda r: norm_s(r['Phase'])

if r[0] else np.nan),

axis=1)

.dropna().tolist())

normality_notes = (survey_normality.isna().apply(

lambda r: [f'n_{phase_int[i]}'

for i in r.index if not r[i]],

axis=1))

sig_s = (lambda i, j: (f"$s^{phase_int[i]}_{phase_int[j]}$: "

f"{i} ⇨ {j} is not statistically significant"))

stat_sig_footnotes = (survey_stat_sig.any().reset_index()

.apply(lambda r: (sig_s(r['Phase'], r['Post'])

if r[0] else np.nan),

axis=1)

.dropna().tolist())

stat_sig_notes = (survey_stat_sig.isna().apply(

lambda r: [f's^{phase_int[i]}_{phase_int[j]}'

for i, j in r.index if not r[i, j]],

axis=1))

cols = ['mean', 'std', 'delta', 'N', '%']

survey_stats = (raw_stats.loc[:, (slice(None), cols)]

.rename({'mean': 'μ', 'std': 'σ', 'delta': 'Δ'}, level=1, axis=1))

survey_stats.footnotes = [f'N={N}'] + normality_footnotes + stat_sig_footnotes

iterator = (normality_notes + stat_sig_notes).iteritems()

# survey_stats.index = [i + (f"$^{{{','.join(n)}}}$" if len(n) > 0 else "")

# for i, n in iterator]

stats_title = ','.join(scores['Survey'].unique())

survey_stats.title = f"{stats_title} Statistics"

survey_stat_sig.columns = [

f'{c[0]} ⇨ {c[1]}' for c in survey_stat_sig.columns]

survey_p_vals = pd.concat([survey_normality, survey_stat_sig], axis=1,

keys=['Normality', 'Significance'])

p_val_title = ','.join(scores['Survey'].unique())

survey_p_vals.title = f"{p_val_title} p-values (p<{alpha})"

return (survey_stats, survey_p_vals)

## Python code effect size calculation

import pandas as pd

def cohens_d(data_1: pd.Series, data_2: pd.Series) -> float:

"""Calculate the Cohen's d effect size for two series

Parameters

----------

`data_1` [pd.Series]

- list 1 which in all cases should be the pre phase

`data_2` [pd.Series]

- list 2 which in all cases should be the post phase

Returns

-------

[float]

- Returns the Cohen's d

Additional Resources

--------------------

Included are the used definition of Cohen's d and the Pooled Standard Deviation

- [Cohen's d](Statistical Power Analysis for the Behavioral Sciences; Second edition; p.66-67)

"""

n1, n2 = data_1.count(), data_2.count()

m1, m2 = data_1.mean(), data_2.mean()

s = ((((data_1 - m1)**2).sum() + ((data_2 - m2)**2).sum()) / (n1 + n2 - 2))**.5

d = (m2 - m1) / s

return d
